# Platforms for the Search for New Antimicrobial Agents Using In Vivo C. elegans Models

**DOI:** 10.32607/actanaturae.27348

**Published:** 2024

**Authors:** A. I. Kalganova, I. E. Eliseev, I. V. Smirnov, S. S. Terekhov

**Affiliations:** Shemyakin–Ovchinnikov Institute of Bioorganic Chemistry, Moscow, 117997 Russian Federation; Department of Chemistry, Lomonosov Moscow State University, Moscow, 119991 Russian Federation; Endocrinology Research Center, Moscow, 117292 Russian Federation

**Keywords:** C. elegans, microfluidics, infection model, pathogens, drug discovery, antimicrobials

## Abstract

Despite the achievements brought about by high-throughput screening technologies, there is still a lack of effective platforms to be used to search for new antimicrobial drugs. The antimicrobial activity of compounds continues, for the most part, to be assessed mainly using *in vitro *pathogen cultures, a situation which does not make easy a detailed investigation of the molecular mechanisms underlying host–pathogen interactions. *In vivo *testing of promising compounds using chordate models is labor-intensive and expensive and, therefore, is used in preclinical studies of selected drug candidates but not in primary screening. This approach does not facilitate the selection of compounds with low organ toxicity and is not suitable for the identification of therapeutic compounds that affect virulence factors. The use of microscopic nematode *C. elegans* to model human infections is a promising approach that enables one to investigate the host–pathogen interaction and identify anti-infective compounds with new mechanisms of action.

## INTRODUCTION

The antibiotic resistance crisis goes hand in hand with the problem of searching for and developing new antibiotics. The first antibiotics were discovered using the principle of screening small compound libraries *in vivo *on animals, such as infected mice and rabbits [1]. This approach was soon abandoned in favor of a more productive, ethical, and convenient one: the testing of antibiotics on pathogen cultures *in vitro *[2]. Almost a century after the discovery of the first classes of antibiotics, the spread of resistance and an acute shortage of new antibiotics forced researchers to look for new high-throughput platforms and return to *in vivo* screening [3].

Currently, there are a number of effective platforms for screening antibacterial drugs active against multidrug-resistant pathogens, biofilms, and intracellular pathogens [3]. However, microbial resistance seems to remain a step ahead of efforts towards modern approaches to the search for and testing of new therapeutic molecules. Antimicrobial activity is for the most part assessed in pathogen cultures *in vitro*, but that hampers any detailed investigation of the molecular mechanisms mediating the host–pathogen interaction.

A new strategy may be searching for molecules possessing alternative mechanisms of action; e.g., compounds that block virulence, stimulate the immune response, or are prodrugs. Such compounds, which are called anti-infectives, as opposed to antibacterials, cannot be identified in conventional experiments on pathogen cultures *in vitro*. To search for them, infections are currently modeled on whole organisms: the nematode *Caenorhabditis elegans*, the fruit fly* Drosophila melanogaster*, and the fish *Danio rerio *[4]. The objective is to identify, by screening large compound libraries, both compounds that inhibit the activity of regulators of virulence factor production in certain pathogens and compounds that activate innate immunity [5].

Screening at the organism level has a number of advantages, the main one being the simultaneous acquisition of data on activity and toxicity, which makes the transition to other models more linear. Off-target effects, complete absorption, physiological distribution, general metabolism, and assessment of early toxicity* in vivo *also help to prioritize the selection of potential candidates [6]. The solution is to use small animals that have a simple biological system for the implementation of natural infection mechanisms in laboratory conditions. The model organism nematode* C. elegans *is suitable for high-throughput screening thanks to its small body size, short life cycle, and easy culture maintenance.

**Fig. 1:**
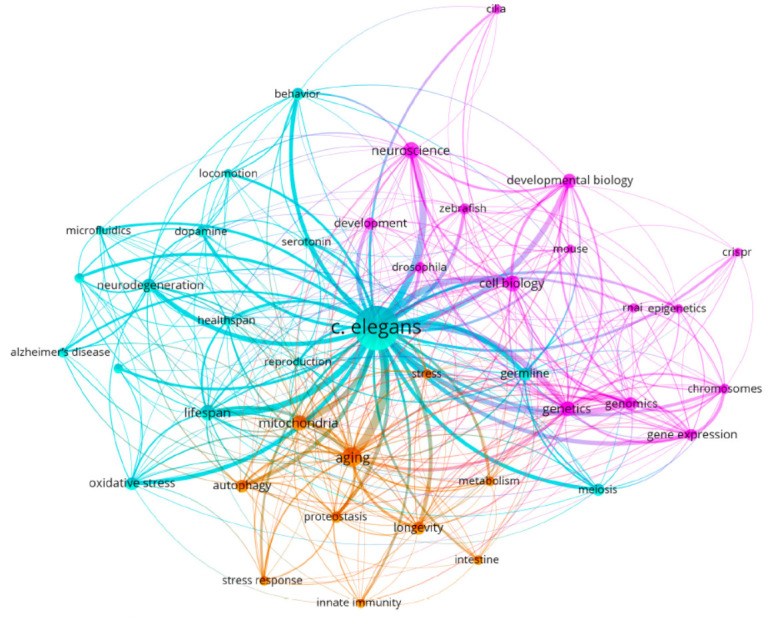
Most popular areas of research with C. elegans. The map was generated using the VosViewer software; the search was performed using data from the PubMed database, from 2018 to 2023

*C. elegans* is a popular model used in genetic and physiological studies (*Fig. 1*). Recently, this organism has found increasing importance as a model for studying the mechanisms of host–pathogen interactions at the systemic level [3, 4].

The microscopic nematode *C. elegans *was first used to screen antibiotics in an infection model in 2006 [7]. The very first study discovered several compounds that suppressed development of the infection but did not kill the pathogenic bacteria. This points to the ability of such an *in vivo *model to identify molecules with alternative mechanisms of action. Soon, *C. elegans* was being shown to be well suited for modeling many human infections, both bacterial and fungal ones [8], and for studying intracellular infections [9] and biofilms [10].

The results of screening and identification of antimicrobial compounds using *C. elegans *have been published [11, 12]. Several research groups that have developed *in vivo *infection models and technologies for screening chemical libraries in *C. elegans *have identified a number of promising antimicrobial molecules using this system. In particular, a low-molecular- weight compound was discovered that protects the nematode from a *Pseudomonas aeruginosa *infection via the activation of innate immunity [13]. In a resistant *Staphylococcus aureus *infection model, a new class of retinoid antibiotics (CD437 and analogs) effective against bacterial persister cells was discovered [14].

The nematode *C. elegans *is a simple host model for studying the interactions between the innate immune system of animals and various pathogens [15]. Extensive genetic and molecular tools are available for *C. elegans*, which facilitate the indepth analysis of host defense system components shared with mammals, and pathogen virulence factors.

Those investigations of the *C. elegans *response to bacterial infections revealed that the immune system of this organism uses evolutionarily conserved signaling pathways and synthesizes a number of effector molecules, some of which are also conserved (e.g., p38 MAPK signaling pathway) [16]. Despite having demonstrated immune responses to infection, the precise pattern recognition receptors in *C. elegans* remain to be identified.

*C. elegans *is the first multicellular organism with a fully sequenced genome. A high degree of similarity (60–80%) between many nematode genes and human ones has been established using bioinformatic approaches [17], which makes *C. elegans* a valuable model test object for toxicity studies [6]. As a result, the nematode *C. elegans *has become an instrumental model through which to understand the mechanisms of molecular pathogenesis of many human diseases. The innate immunity of *C. elegans *has become a subject matter in the study of immune defense and the role of cellular stress in the organism’s response to infection, in particular in modeling gene activation in response to infection [18].

*C. elegans* **BACTERIAL INFECTION MODELS**

*C. elegans *can be infected with a selected pathogen by substituting its usual laboratory food source; e.g., with the *Escherichia coli *strain OP50, which is relatively non-pathogenic for this nematode. The bacterial environment is natural for nematodes [19]. The use of heat-killed *E. coli *bacteria is no more advantageous than the use of live bacteria, because thermal destruction makes such food unattractive for nematodes and, also, because they no longer contain all the nutrients essential for nematodes’ normal development. In the control group of the *C. elegans *experiment, the use of bacteria killed by UV radiation is believed to be optimal [20]. Nematodes display behavioral reactions that develop in response to a bacterial pathogen [21]. Bacterial evasion and innate immune response are two ways in which *C. elegans *respond to pathogens [22].

There are various possible ways how the active substance can act in the model under consideration: direct killing, alteration of the nematode behavior, reduced pumping of the neuromuscular pump that joins the mouth to the intestine, activation of innate immunity, and influence on the quorum sense in bacteria; i.e., suppression of biofilm formation and transition to a chronic infection.

**Table 1:** Main nematode infection protocols

Protocol	Main characteristics
Slow killing	The destruction mechanism based on an infection-like process includes identification and proliferation of the pathogen in the intestine, with biofilm formation, and investigation of the suppression of bacterial pathogenesis
Fast killing	The main role is played by phenazine-1-carboxylic acid, which is extremely toxic to cells in an acidic environment
Liquid killing	Released endotoxins provide hypoxic conditions

To date, there are standard protocols for infection and analysis of the bacterial effects on the vital activity of nematodes: e.g., a quantitative assessment of the bacterial load in *C. elegans *ISO 10872 [23, 24, 25, 26, 27]. Slow-killing models an infection-like process. The protocol uses agar, which is difficult to automate. In this case, it should be taken into account that the optimal temperature for nematodes to be maintained is 25°C; i.e., bacteria, when eaten by worms, continue to grow nonetheless. After the use of nematodes as a model organism for the investigation of bacterial infections had been demonstrated as fitting, liquid killing and fast killing protocols were also developed (*Table 1*). To date, these are the main protocols used [28].

The fast-killing mechanism is mainly focused on the action of the toxins in the medium. The liquid protocol does not provide for stable intestinal colonization or a normal life cycle for the nematode (difficult defecation, long egg retention, and, as a result, the formation of a ‘bag of worms’ phenotype). For example, infection with *P. aeruginosa *is accompanied by the secretion of pyoverdin, which is necessary for the replenishing of the intracellular iron pool in the bacterium. This siderophore, along with other substances, is absorbed by *C. elegans* from the liquid medium [29, 30]. After entering the host, pyoverdin gains access to ferric iron and removes it [31, 32], which leads to rapid cellular death of the nematode. Most protocols focus on the total toxic load, whereas the level of bacterial load in the nematode’s digestive tract is often not analyzed.

***C. elegans *survival analysis**

Many traits and characteristics of the nematode are used to assess the effect of a pathogen or a test compound: lifespan, body curvature and length, pharyngeal pump activity, number of bacteria inside the body, fat storage, vulva integrity changes, and progeny number. Also, stress assays are performed: the effects of thermal, acoustic, and oxidative stress are analyzed; changes in host gene expression and fluorescence initiated by the triggering of a certain signaling pathway are assessed; and accumulation of certain proteins is measured [33, 34]. The estimate of mean survival time of worms exposed to a certain bacterial isolate corresponds to the measure of bacterial virulence [35]. In such experiments, the 50% lethal time (LT50) is determined [34].

The lifespan can be determined in both solid and liquid media. A typical protocol involves counting live and dead worms from an initial synchronized population over a particular period of time [6]. Live and dead worms are counted in response to poking with a platinum wire, shaking, or exposure to light or based on the fluorescence signal of a vital dye (in liquid media). Upon nutrient deficiency, bacteria can secrete toxic metabolites and endotoxins into the medium. In this case, the survival analysis is multifactorial.

The first study on the use of *C. elegans *to model infections demonstrated that nematodes seeded into the wells of a plate with the culture medium remained viable for at least 14 days [7]. What allows nematodes to retain viability? Apparently, this is achieved thanks to simultaneous transfer of nematodes with bacteria, which are a feed source for the worms, as well as a sufficient amount of the nutrient medium to maintain the bacterial population.

Work with *C. elegans *began with detailed genetic typing, which later, together with the relative simplicity and convenience of experiments with this nematode, made this species a model [35]. Investigation of the microorganism–host interaction in the *C. elegans *model may ultimately provide information on how microbes affect the nervous system function in more complex animals [36], because a very close similarity between data obtained in mice and nematodes has been repeatedly demonstrated [37, 38].

The results obtained to date indicate the importance of accumulating a large body of homogeneous data. There exists a methodology for massive, simultaneous observation of nematodes, which may help in conducting complex genetic and behavioral studies, increasing the number of phenotypes that can currently be detected using larger numbers of simultaneously observed organisms [39]. However, this approach, although increasing the significance of the results, does not intensify the testing process.

**Social behavior**

Nematodes feeding on bacteria on agar often engage in communal feeding, which also influences the amount and rate of bacterial feeding [21]. Wild-type *C. elegans *isolates aggregate and feed in groups when grown in laboratory conditions, while the N2 laboratory strain consists of solitary feeders. The most potent hypothesis for why wild-type isolates aggregate is that aggregation allows the avoidance of high-oxygen environments. Pathogenic bacteria can infect *C. elegans *by attaching themselves to the cuticle, and collective feeding may mitigate the risk of infection by reducing surface exposure to bacteria [40]. Additionally, the developing phenotype is influenced by the presence and concentration of ascarosides, which are important small-molecule signals in nematodes. Different combinations of ascarosides mediate different phenotypes, and even small differences in their chemical structure are often associated with highly altered activity profiles in nematodes [41].

**Probiotics**

*C. elegans *has turned into a useful model for studying innate immunity in terms of microbiota–host interactions [42]. The molecular pathways initially triggered by pathogens are highly conserved in a large variety of organisms, from insects and nematodes, to mammals [43].

Animal probiotics can include diverse members of the microbiome, in particular *Bacillus subtilis*,* Lactobacillus spp*., *Pseudoalteromonas spp*., etc. [44, 45, 46]. The mechanisms of disease control by probiotics include enhanced immune response, competitive adhesion, pathogen antagonism, and disruption of the QS system. An important way in which probiotics can protect the host from pathogenic bacteria is to reduce bacterial colonization of the host gut and inhibit subsequent bacterial growth, which maintains the overall balance of the host gut microbiome composition [47]. Although many studies have shown that probiotics exhibit antibacterial and antifungal activity, their main mechanism of action is to reduce zoonotic pathogen infection-induced toxicity, either by displacing pathogens or by neutralizing toxic molecules [48].

The *C. elegans *model can be used not only in tests of antimicrobial drugs, but also in the search for new probiotics [49, 50, 51]. The relevance of *C. elegans *as a model organism in probiotic studies and elucidation of various molecular mechanisms is associated with highly conserved signaling pathways similar to those in higher mammals [51, 52].

**The bacteria used to infect *C. elegans***

**Table 2:** Examples of last-decade studies with testing of different bacterial pathogens in the C. elegans infection model

Bacterial pathogen	Test antibacterial compound	Protocol type	Reference
E. coli	-	Liquid killing	[104]
	Bacteriophages		[87]
A. baumannii	Curcumin, flavonoids	Liquid killing	[105]
	–	Slow killing	[106]
	AMP library	Liquid killing	[86]
M. nematophilum	–	Dar phenotype formation	[107], [108]
S. typhimurium	–	Liquid killing	[109]
S. aureus	Amoxicillin	Liquid killing	[110]
	P. guajava leaves extract	Liquid killing	[111]
	Resveratrol, econazole, paraquat	Slow killing	[74]
	AMP library	Liquid killing	[86]
	Panchgavya	Liquid killing	[50]
	Lactobacillus curvatus BGMK2-41	Slow killing	[43]
S. gordonii	–	Slow killing	[112]
L. monocytogenes	–	Slow killing	[113], [114]
P. aeruginosa	P. guajava leaves extract	Liquid killing	[111]
	Combination of linezolid and polymyxin B	Liquid killing	[73]
	Peonol	Liquid killing	[115]
	AMP library	Liquid killing	[86]
	Bacteriophage	s Liquid killing	[87]
	B. megaterium and P. mendocina	Slow killing	[52]
	Gentamicin	Slow killing	[116]
	Holothuria atra	Liquid killing	[117]
	Lactobacillus curvatus BGMK2-41	Slow killing	[43]
S. marcescens	P. guajava leaves extract	Liquid killing	[111]
S. pyogenes	P. guajava leaves extract	Liquid killing	[73]
C. violaceum	P. guajava leaves extract	Liquid killing	[73]
B. thuringiensis	-	Liquid killing	[118]
	Lipopeptide thumolycin		[119]
B. anthracis	–	Slow killing	[120]
E. faecalis	AMP library	Liquid killing	[86]
	–	Slow killing	[55]
E. faecium	–	Slow killing	[55]
B. cepacia	–	Slow killing	[121]
E. cloacae	–	Slow killing	[122]
	Bacteriophages	Liquid killing	[87]
B. cereus	Carvacrol	Slow killing	[123]
H. pylori	Fucoidan extract	Slow killing	[124]
S. pyogenes	Biflavonoid fukugiside	Liquid killing	[125]
C. diphtheriae	–	Dar phenotype formation	[126]
C. violaceum	Peonol	Liquid killing	[115]
K. pneumoniae	Bacteriophages	Liquid killing	[87]
	Range of antibiotics		[127]

The effects of gram-negative *P. aeruginosa *and gram-positive *Staphylococcus aureus *have been well studied in nematodes [53, 54]. But recently, investigation of pathogenesis and biofilm formation has enabled the application of existing approaches to pathogen species (*Table 2*).

*C. elegans *is capable of mounting a specific response to bacterial pathogens at the transcriptome level. However, various bacterial pathogens, including *Enterococcus faecalis*, *Enterococcus faecium*,* Staphylococcus aureus*, *Serratia marcescens*, and *Photorhabdus luminescens*, also activate the expression of the same innate immune genes [55]. All of these bacterial pathogens cause colonization and bloating of the *C. elegans *intestinal lumen. Colonization with *P. aeruginosa *results in the activation of immune response genes and pathogen avoidance responses in *C. elegans*. Intestinal bloating caused by microbial colonization activates immune response genes and neuroendocrine pathways, inducing an avoidance response [56]. The ability to reveal specifically the regulated genes and pathways in the host or pathogen may help identify the novel metabolites produced by bacteria that affect host physiology [57].

**Colonization by multiple bacterial species**

The gastrointestinal microbiota is a complex microbial ecosystem. The influence of particular microorganisms on host signaling pathways can vary. There is growing evidence that genetic host variability determines the abundance of specific taxa living in the body [58]. For example, the possibility of co-culture of several pathogens in the nematode intestine was shown in [59]: two [60] or three [58] bacterial species and even transfer of the human intestinal microbiome [61]. Such experiments are performed to elucidate the role of interspecies interactions in the formation of host-associated microbial communities. Experimental bottom-up microbial ecology is a tool for studying the dynamics of bacterial gut communities in a model organism *C. elegans*, allowing us to elucidate the role of interspecies interactions in the combined microbiome–host system and bacterial competition within an *in vivo *environment [62].

## DRUG DELIVERY

**Toxicological tests**

One of the first areas of testing compounds using* C. elegans *as a model was toxicity testing in a liquid culture. Such tests were initially performed using the live/dead assay, plotting dose–response survival curves [63], then using behavioral tests [63, 64], and assessing specific phenotypes [65, 66, 67]. Later studies have demonstrated that the nematode is an organism suitable for studying toxicity and assessing the efficacy of some medicinal compounds.

Rapid toxicity tests are still used to this day [68, 69, 70]. Often, this model is used to test the toxic activity of bactericidal drugs with the efficacy proven *in vitro* [71]. In this case, not only solutions of synthetic compounds [72], but also natural extracts [73], nanoparticles [73, 74], and natural isolates [75, 76] are tested. This model was exploited to figure out a way to reduce the toxicity of a cryoprotectant applied in transplantation [77].

Screening of compounds using *C. elegans *enables a preliminary assessment of drug toxicity, which allows one to exclude compounds toxic to the host at an early stage, whereas *in vitro *testing identifies only bactericidal or bacteriostatic compounds [78]. Nematodes have been used in high-throughput drug screening to assess both toxicity and efficacy, and this screening approach has been commercialized by several companies (Nagi Bioscience, *InVivo *Biosystems, Magnitude Biosciences) [79].

**Drug screening**

In the nematode infection model, there is a limited choice of approaches for the delivery of test compounds: delivery by mixing a solution of the active agent with nematodes in a liquid nutrient medium [80] or adding to the solid medium [81]; delivery by mixing a solution of the active agent with a bacterial nutrient source (including labeling of bacteria) [82, 83].

If we consider such a method of delivery of the active agent as its packaging into micro- or nanoparticles, then the delivery will be one of the simplest ones, but an effective strategy that mimics the natural feeding of nematodes by the swallowing of bacteria- like microparticles. When the food content in the environment is low, nematodes can reduce the level of pharyngeal pumping to avoid ingesting nonnutritional particles; however, at high particle levels, many foreign particles still get inside worms [84]. This method provides targeted delivery of the active agent to the pathogen, avoiding toxic effects on tissues. Similar methods are also useful for assessing the pharmacokinetics of natural compounds [32]. Although nematodes are a promising model system for screening antimicrobial compounds, they are still far from fully reproducing mammalian biology. For example, nematodes have an effective detoxification system that can limit potential identification of compounds that act through the modification of host defense systems [85].

Many different classes of compounds have been tested for toxicity and efficacy using the *C. elegans* model [23, 24, 25, 26, 27]. The widest range of diversity comes with antimicrobial compounds, because the possibility to induce an infectious process in *C. elegans *using a variety of microorganisms provides for a large number of test pathogen–antimicrobial agent combinations, even without the simultaneous use of several drugs.

*C. elegans *lacks professional immune cells. Due to the lack of an adaptive immune system, this nematode relies solely on its innate immune defense to cope with a pathogen attack. In response to external stimuli, a cascade of reactions is triggered, which leads to the release of antimicrobial peptides (AMPs). AMPs are biologically active molecules produced by a variety of organisms and are an important component of nematode’s innate immune response. For example, the effect of a small AMP library was tested and data on the efficacy of cecropin derivatives were collected. They were consistent with generally approved data [86].

This approach was first applied in a *C. elegans* model for a relatively low-throughput screening of 7,136 synthetic compounds and natural product extracts for activity against the opportunistic human pathogen *Enterococcus faecalis *[7]. Of these, 12 compounds were shown to provide host protection *in vivo* at concentrations significantly lower than the minimum inhibitory concentrations *in vitro*.

*C. elegans *infection models allow high-throughput screening of new anti-infective molecules. Such molecules may be used as probes to identify new mechanisms of bacterial pathogenesis [12]. These models may also be used to test the antimicrobial activity of bacteriophages before large-scale preclinical studies in mice [87]. The production of nematode biosensors that respond to changes in the intestinal microbiome composition seems promising. A biosensor for analyzing the host–microbiome interaction in the digestive tract was created in [62].

There are studies devoted to the search for new compounds using bioinformatic methods in the* C. elegans *model. For example, the effect of some compounds on the nematode lifespan was predicted using the DrugAge database [88]. This approach may be translated into a prediction of the effect of compounds and pathogens on the nematode by creating a database of their mechanisms of action. Another method to analyze the response of nematodes is optogenetics. The use of optical methods enables quantitative monitoring of the metabolism of intestinal bacteria to assess the local and systemic effects of test compounds on nematode health [89].

**Microfluidic technologies as a transition to personalized medicine**

The possibility to manipulate single live *C. elegans* nematodes using microfluidics [76] is widely used in behavioral studies and microscopy. Studies in this area are focused on the search for antibiotics using medium- sized chemical libraries; for this purpose, 384-well plates are suitable. The development and behavior of* C. elegans *are studied using a variety of microfluidic technologies [78].

The use of any microfluidic chip ensures low consumption of synthesized bioactive molecules, such as AMPs, as well as targeted delivery of potential drugs in a small volume of liquid. The use of microfluidic trap technologies excludes the mutual influence of nematodes. Therefore, the natural development of elaborated approaches would be the use of high-throughput microfluidic screening technologies, which enable an analysis of large libraries of active compounds.

The existing platforms are divided mainly into four types: (i) platforms for monitoring lifespan and aging [90], (ii) platforms for screening toxicity and pathogenesis, (iii) platforms for studying neurobiological phenomena and behavioral tests [91], and (iv) platforms for drug discovery. Most of the developed microfluidic chips are aimed at solving the problems of sorting and studying the larval stages of nematodes.

The significant advantages of microfluidics have led to the development of devices for survival curve measurements. Microfluidic encapsulation of nematodes in single compartments was shown not to affect their lifespan [92]. Similar developments in the field of microfluidic technologies enable a transition from labor-intensive experiments on Petri dishes to automated and productive platforms for candidate selection. Metabolic by-products accumulate in worms and bacteria, and the biological state of bacteria changes in response to stress factors, which can have a secondary effect on worms. Although this effect can be minimized by repeated transfer of animals to new dishes, physical manipulations can lead to additional stress and partial loss of the population. The possibility to accurately and quickly control the environment is one of the many advantages of microfluidic devices [93]. There are also a number of responses to starvation as a stress factor. One such response is the cessation of egg-laying in adulthood. Cessation of egglaying leads to matricidal internal hatching of progeny that is subsequently used by the mother as a food source. Such data are usually censored during statistical processing [94].

The use of microfluidic technologies solves such automation problems as (i) programmable control of fluid flows, handling small volumes of active compounds; (ii) uniform dosing of nematodes by volume; (iii) compartmentalization, in particular by sorting, and phenotypic profiling of individuals; (iv) long-term culturing under relatively constant environmental conditions; and (v) real-time monitoring, tracking multiple checkpoints.

A microfluidic chip with progeny filtration can be used to investigate aged populations without chemical sterilization (FUDR) or frequent plate-to-plate transfers, thereby avoiding the use of sterile strains [95]. Immobilization of single nematodes in a channel can be an excellent way to score high-resolution, real-time images [96].

The main drawback of many devices is that* C. elegans *swims in specially designed chambers, like liquid cultures in multi-well plates. Physiologically, swimming in a liquid culture is more energy consuming than crawling and extends the sleep period, which complicates the phenotyping procedure [97]. While *C. elegans *larvae exhibit quiescence during lethargus, adult worms occur in quiescence only in a few situations; e.g., after several hours of swimming or after exposure to extreme environmental conditions. In a broader context, sleep induced by flow events is defined as a behavior in which experimentally controlled external stimuli strongly influence the animal’s transition speed between behavioral states.

Swimming and crawling worms exhibit significantly different gene expression profiles and lifespans [98]. Therefore, it is assumed that the results obtained using devices in which worms crawl rather than swim are best comparable to those obtained in a solid medium. The problem of immobility in many individuals can be overcome by using light to stimulate arousal and movement [99].

The introduction of microfluidic approaches to advanced visualization of bacterial colony dynamics and digestion kinetics *in vivo *opens the way to increased information content, throughput, and versatility of the methods aimed at assessing the interactions between microbiota and the *C. elegans *gut. Microfluidic platforms for parallel on-chip studies are based on feeding worms with different bacterial strains and/or applying antimicrobial compounds [100]. The immune response was measured by expression of the immune response gene *irg-1* and was used to monitor expression changes upon exposure to the pathogenic bacterial strain *P. aeruginosa* [101]. The most common feature of such platforms is real-time phenotypic analysis of individuals and generation of survival curves from the data obtained [102].

Microfluidic technology that enables the study of bacterial pathogenesis was demonstrated in the Celab system [102]. The technology combines the capabilities of other devices to perform high-throughput monitoring, long-term microfluidic incubation of worms, individual tracking, and semi-automated measurements with progeny washing and food replenishment.

Therefore, microfluidics enables personalized phenotyping because microfluidic chips are able to collect individual responses throughout the worm’s life [103]. Modern microfluidic systems exclude the need for repeated manual transfer of adults during survival tests, progeny sorting, or avoidance of swimminginduced stress throughout the life of fluid-grown animals. Therefore, the overall number of censored worms is reduced [93].

## CONCLUSION

The *C. elegans *infection model can be empirically used as a host–pathogen system to assess the virulence of a new pathogen in studies of the innate immune response. Most of the studies of intestinal infection in *C. elegans *have been performed using a monobacterial culture. However, under natural conditions, the microbiome is represented by a complex consortium of microorganisms. Thus, further research on co-culture of several species is needed.

A logical continuation of the development of the technologies discussed in this review will be creating a microfluidic device that provides the nematode infection stage, followed by testing libraries of potential anti-infective compounds in infected individuals. The creation of such a device is based on the possibility to trigger a stable invasive infection in the nematode gut, as well as in a targeted way deliver test compounds and monitor their effects. Since microfluidics is scalable and adaptable, a microfluidic device may be used not only for basic research of pathogenesis, but also for high-throughput screening of candidate molecules.

A promising area is the combined use of the proposed platform for infection and screening on *C. elegans* and the technology of synthetic libraries of antimicrobial peptide biodiversity. The field of antimicrobial peptide development suffers from a lack of a high-tech tool for high-throughput synthesis and testing of candidate peptides. Formation of synthetic microbiota of antimicrobial peptide producers in *C. elegans* would fill this gap.

## Abbreviations

AMP: antimicrobial peptide
MIC: minimum inhibitory concentration
QS: quorum sensing
